# Mental health problems among transition-aged youth with physical disabilities: an initial evaluation

**DOI:** 10.3389/fresc.2023.1069464

**Published:** 2023-05-05

**Authors:** Amanda Amalfi, Jia Yin Li, Vanessa Théberge-Lamoureux, Carmen Tang, Emilie Rinaldi, Pranamika Khayargoli, Dana Anaby

**Affiliations:** ^1^ School of Physical and Occupational Therapy, McGill University, Montreal, QC, Canada; ^2^ Centre for Interdisciplinary Research in Rehabilitation of Greater Montreal (CRIR), Montreal, QC, Canada

**Keywords:** adolescence, mobility restriction, transition into adulthood, behavioral emotional problems, young adults

## Abstract

**Aim:**

Decreased participation and complex transitions into adulthood among youth with disabilities may impede their well-being. To advance knowledge on the co-occurrence of mental health problems and physical disability, this brief report describes the frequency of mental health problems, measured by the Behavior Assessment System of Children (BASC-3), among transition-aged youth (14–25 years) with physical disabilities and examines the association between mental health problems and sex, age, and number of functional issues.

**Methods:**

Thirty-three participants completed a demographic questionnaire and the BASC-3. Frequency of BASC-3 scales falling within 3 categories: “within norms”, “at risk”, and “clinically significant” were described. Crosstabs and Chi-square tests were used to examine the association between BASC-3 scales and sex, age (< and ≥ 20), and number of functional issues (< and ≥ 6).

**Results:**

Overall, “somatization”, “self-esteem”, “depression” and “sense of inadequacy” were the most common subscales being at risk. Participants with a higher number of functional issues (≥6) were more likely to fall within “at risk” or “clinically significant” categories across 20 (out of 22) BASC-3 scales, and female participants tended to fall more within “at risk” or “clinically significant” categories for 8 of BASC-3 scales. Younger participants (<20) were ranked in the “at risk” or “clinically significant” categories for 7 scales.

**Conclusions:**

Findings lend further support for the occurrence of mental health problems emerging in youth with physical disabilities and highlight initial trends especially across functional levels. Further investigation of such co-occurrences and the factors that affect their development is needed.

## Introduction

Children and youth with physical disabilities often experience lower levels of participation compared to their peers without physical disabilities (1–4). Decreased opportunities to participate in activities and build relationships among individuals with disabilities often result in social isolation, decreased quality of life and higher prevalence of mental health problems (5–7). Examples of barriers to their participation include stigma, negative attitude towards disability, lack of inclusivity, limited opportunities and resources addressing the specific needs of this population (8, 9).

Along with decreased participation, youth with physical disabilities also experience more complex transitions into adulthood that may adversely affect their quality of life and well-being. Adolescence and young adulthood are marked by pronounced psychological and physical changes (10). Mental health problems or initial subclinical symptoms are likely to appear during this sensitive period, causing psychosocial and participation pattern disruptions (11, 12). Moreover, the sudden termination of children's healthcare services, as well as lack of continuity and guidance into new services can add further complexity into the transitioning process of this population (13, 14). Furthermore, clinicians in the field of physical rehabilitation often tend to focus mainly on the youth's physical impairments, and their psychosocial functions, such as their mental health, typically may receive little attention (15). Emerging/early symptoms of mental health problems can deteriorate if left untreated during adolescence (16).

Evidence on the co-occurrence of physical disabilities and mental health problems is emerging. A meta-analysis of 8 studies among children and youth with cerebral palsy conducted by Downs et al. (15) suggests that mental health symptoms are common. Similarly, a recent scoping review by Lal et al. (17) indicated that most studies examining such co-occurrences pertained to youth and young adults with cerebral palsy, with fewer studies including other physical conditions such as juvenile arthritis and spina bifida. In addition, the most common age range across studies was 13–17 (17). Thus, examining co-occurrences in a broader age range that covers the transition-aged period, across various types of physical disabilities, is needed. Using psychometrically sound measures that can broadly assess aspects of mental health problems in terms of individual's behaviors and emotions, is warranted. An example of such measure is the Behavior Assessment System of Children-Third Edition (BASC-3). The BASC-3 is a multicomponent instrument intended for individuals aged 2–25, that allows for evaluation of numerous behaviors pertaining to a range of mental health components and includes clinical norms by age and sex (18).

To better understand the occurrence of mental health problems among transition-aged youth living with physical disabilities, this brief research report aimed to describe the frequency of mental health problems, as measured by the BASC-3, among youth aged 14–25 years with physical disabilities who experience different levels of mobility restriction in a large Canadian province where both English and French are common spoken languages. It also aimed to describe the frequency of mental health problems according to sex, age, and complexity of youth's condition in terms of number functional issues among this population.

## Material and methods

### Setting and procedure

This brief research report presents baseline results (in terms of the presence of mental health problems) of a clinical trial that examined the effect of an intervention aimed to improve participation in leisure community-based activities on outcomes at the body function level using the specific subscales of the BASC-3 among other motor-related outcomes. As such, the present study is cross-sectional in nature. A total of 33 youth aged 14 to 25 years old (mean age: 20.24) were included from both sexes who spoke either English or French. Youth with a physical disability, presenting with restricted mobility (requiring adaptive equipment, physical aid, or external support/supervision), as well as multiple diagnostic categories were included. Participants were excluded if they were in their first year of recovery from severe brain injury or orthopedic surgery or if they were recovering from botulinum toxin treatment 6 months prior to the study or anticipated during the study. Youth with neurodegenerative disorders and/or significant intellectual delays were also excluded, as the latter had the potential to impact self-completion of any necessary forms and assessments.

Convenience sampling was used to recruit youth from three large rehabilitation centers/hospitals in the Greater Montreal area. Consent forms were obtained online *via* Research Electronic Data Capture (RedCap) from all participants followed by the completion of a demographic questionnaire. The full BASC-3 was then completed online by the youth through the Q global platform (18) and generated encrypted summary reports were stored in a secured platform. Ethics approval was obtained by the Centre for Interdisciplinary Research in Rehabilitation of Greater Montreal (CRIR).

### Measures

Behavior Assessment System for Children- Third Edition (BASC-3). The BASC-3 was used to measure behaviors and emotions that pertain to aspects of mental health. It is a multicomponent instrument that takes self-reported measures to analyze behavioral and emotional aspects of children and youth (2–25 years old) (18). Two different self-reported forms of the BASC-3 were used in this study: the adolescent form (for ages 12–21 years) and the college form (for ages 18–25 years), that provide comparable results. This measure contains 175 different items pertaining to certain behaviors or emotions, that are categorized into 16 clinical and adaptive subscales (e.g., anxiety, social stress) from which content and composite scales are generated (see Table 1 for details). Each BASC-3 assessment takes 20–30 min to complete. Youth are asked to rate if and how often they experience items on either a True/False scale, or a 4-point Likert scale with options: “Never”, “Sometimes”, “Often” and “Almost always”. Examples of items include “I feel sad”, “I worry a lot of the time” or “I am left out of things”, among others. The Q-global platform is used for immediate scoring and reporting of the completed adolescent and college self-reported forms (18). Responses from each item generate a score, which are summed to obtain a raw score per subscale. T scores and percentiles are generated for each subscale using the raw scores. The T-score is then placed within one of three categories: “within norms” (T-scores < 59), “at risk” (T-scores ranging from 60 to 69 for clinical scales and 31–40 for adaptive scales) or “clinically significant” (T-scores ≥70 for clinical scales and ≤30 for adaptive scales). The BASC-3 is a valid and reliable tool; evidence of internal consistency ranges from 0.71–0.97 for ages 12–25 years. Test-retest reliability ranges from 0.72–0.90 across scales (18). Evidence of correlations between the BASC-3 with various measures of behavior has also been examined, where for example, moderate or high correlations between self-reported clinical scale scores of the BASC-3 and similar Achenbach System of Empirically Based Assessment Youth Self-Report Form syndrome scale scores were seen, supporting its construct validity. Factor analysis was also confirmed by grouping of scales into composites, supporting the domains of the BASC-3 and, hence, its structural validity. The BASC–3 also offers a number of scales that help detect threat to validity (18).

**Table 1:** Types of self-reported BASC-3 scales.

BASC-3 Scales	BASC-3 Scale components	Adolescent form	College form	BASC-3 Scale description
**Composite Scale**				
**School problems**		**✓**		Composite that reflects school problems
	Attitude to school	**✓**		Feelings regarding school such as liking or disliking going to school, alienation, dissatisfaction
	Attitude to teachers	**✓**		Feelings regarding their teacher. Seeing them as overwhelming, unfair, too demanding.
	Sensation seeking	**✓**	**✓**	Tendency to search for new experiences, feelings and the readiness to take risks
**Internalizing problems**		**✓**	**✓**	Tendency to be compliant to demands and monitor their own actions
	Atypicality	**✓**	**✓**	Having unusual thoughts and perceptions that can be perceived as odd or different from others.
	Locus of Control	**✓**	**✓**	Extend of control the person has over events occurring in their life
	Social Stress	**✓**	**✓**	Level of comfort maintaining relationships with others and in social events
	Anxiety	**✓**	**✓**	Worrying, nervousness, and/or inability to relax over impending or anticipated events
	Depression	**✓**	**✓**	Feeling sad, being misunderstood, loss of interests and/or feeling that life is getting worse and worse
	Sense of Inadequacy	**✓**	**✓**	Level of satisfaction with their ability to perform a variety of tasks or reach their goals
	Somatization	**✓**	**✓**	Experiencing numerous health-related problems (headaches, sore muscles, stomach ailments and/or dizziness) related to psychological or emotional factors expressed in physical symptoms
**Inattention/hyperactivity**		**✓**	**✓**	Items associated with ADHD symptomatology
	Attention Problems	**✓**	**✓**	Ability to maintain necessary levels of attention or concentration to complete tasks.
	Hyperactivity	**✓**	**✓**	Maintaining a level of self-control that is appropriate in situations and is similar to the levels displayed by others their age
**Emotional symptoms index**		**✓**	**✓**	Global indicator of emotional disturbance regarding the thoughts and feelings of the individual
**Personal adjustment**		**✓**	**✓**	Level of adjustment and coping skills regarding life situations
** **	Relations with Parents	**✓**	**✓**	Positive bond or relationships between the parents and child
** **	Interpersonal Relations	**✓**	**✓**	Level of comfort to connect, establish and maintain relationships with others.
** **	Self-Esteem	**✓**	**✓**	Level of self-worth and personal value reported compared to others their age
** **	Self-Reliance	**✓**	**✓**	Ability to know your own capacity, make decisions and depend on yourself
**Additional Scale**				
	Alcohol abuse		**✓**	Difficulty controlling their alcohol consumption and it can negatively impact their life
	School maladjustment		**✓**	Uneasiness or difficulties to meet the expectations in the school context
**Content Scale**				
Anger control		**✓**	**✓**	Difficulty to self-control and regulate their emotions during situations resulting in impulsivity, anger and/or irritation
Mania		**✓**	**✓**	Tendency to experience excessive variation changes in mood and activities
Test Anxiety		**✓**	**✓**	Irrational worry or fear of taking school tests regardless of the person's knowledge or level of preparation for the test
Ego Strength		**✓**	**✓**	Ability to maintain their identity and sense of self in face of adversity

Demographic questionnaire. The demographic questionnaire contained 14 different questions created by the research team. Listed questions were related to sex, age, type of community, spoken languages, living situation, education, engaged activities, functional issues, health conditions, rehabilitation services received (e.g., occupational therapy, physical therapy, and speech language pathology) and mental health services received (e.g., counseling). More specifically, the current study looked at age, sex, and functional issues within the questionnaire. Age was noted in years and sex was categorized as male or female. To capture the complexity of participants' condition, as used in a study by Anaby et al. (19) to predict participation outcomes, a list of 11 functional issues were presented to the participants, in which they reported if they had either “No problem”, “Little problem” or “Big problem”. Participants were considered to have one functional issue if they checked off either “Little problem” or “Big problem”. The number of functional issues ranged from 0 to 11. Functional issues looked at motor, cognitive, learning, and sensory components. Examples of functional issues included “moving around”, “using your hands to do activities”, “managing emotions”, “learning new information”, etc.

### Data analysis

Descriptive statistics were used to report the frequency (presented in percentages) of observed mental health problems falling in categories “within norms”, “at risk”, and “clinically significant” for the entire sample. A bar chart was created to illustrate the percentage of participants who ranked “at risk” and “clinically significant” for each BASC-3 scale. A fourth category named “at risk and above” was created by combining “at risk” and “clinically significant” categories. To examine the association between the presence of mental health problems and variables of sex, age and number of functional issues, crosstabs and Chi-Square or Fisher Exact tests were performed. To do so, the data was divided into 2 subgroups for variables of sex (female, male), number of functional issues (<6 and ≥6) and age (<20 and ≥20) based on the median. Chi-Square tests were performed to generate 2 × 2 crosstabulation tables to explore the relationship between 2 BASC categories (“within norms” and “at risk and above”) of each BASC-3 subscale and the 2 categories of each of the 3 variables (sex, number of functional issues, age). The significance of association between subscales and the 3 variables were determined using the *p*-value of Pearson Chi-Square, but when Chi-Square's assumption of cell counts with less that 5 or less than 20% was violated, the *p*-value of Fisher's Exact Test was used (20, 21). Trends between subgroups of each variable were identified using the expected and observed count of each subscale category, allowing us to examine the likelihood of specific subgroups to fall within the two BASC categories. When analyzing the association between the BASC-3 subscales and the three variables, the following subscales were excluded because they were not present in both adolescent and college forms: alcohol abuse, school maladjustment, attitude to school, attitude to teachers and school problems. All analysis was done using SPSS version 28. Results were deemed statistically significant at *p* < 0.05.

## Results

Thirty-three youth aged 14–25 years (mean 20.24; SD = ±3.05; median = 20) participated in the study. Nineteen youth reported types of services received; 5 (26.4%) of which reported not receiving neither physiotherapy, occupational therapy, counseling, nor speech-language pathology and 14 (73.6%) participants responded to receiving at least one of these services. The number of services received ranged from 0 to 4 (median and mode were 1), with occupational therapy being the most common service received. Eight reported receiving counseling. The majority (90%) lived with at least one of their family members and the remaining (10%) lived alone or with their significant other. Youth reported up to 11 functional issues (mean = 5.3; median = 6). The two most frequently reported functional issues were moving around (69.7%) and managing emotions (68.8%). Youth reported 1–6 health conditions (mean = 2.06; median = 1.0). The two most frequent health conditions were orthopedic/movement difficulties (69.7%) and health impairments (24.2%) as detailed in Table 2.

**Table 2:** Demographic characteristics of participants (*n* = 33).

Characteristics	*n* (%)
**Covid**	
Pre-covid	9 (27.3)
Covid	24 (72.7)
**Type of BASC-3 form**	
Adolescent	16 (48.5)
College	17 (51.5)
**Sex**	
Female	20 (60.6)
Male	13 (39.4)
**Type of community (n = 32)**	
Major Urban	15 (46.9)
Suburban	10 (31.3)
Small town	7 (21.9)
**Language**	
English	12 (36.4)
French	13 (39.4)
Other	6 (18.2)
English and French	1 (3.0)
French and Other	1 (3.0)
**Education (n = 30)**	
High School or Less	22 (73.3)
Some college/University or Tech Training (at least one year)	3 (10.0)
Graduated college/University	2 (6.7)
Graduate Degree	1 (3.3)
Vocational Training/Diploma	2 (6.7)
**Engaged Activity (n = 32)**	
Working Part Time/Seasonal	1 (3.1)
Going to School	24 (75.0)
Recovering From Illness or Disability	4 (12.5)
Other	3 (9.4)
**Rehabilitation Service**	
Counseling (n = 16)	
Yes	8 (50.0)
No	8 (50.0)
Occupational Therapy (n = 19)	
Yes	10 (52.6)
No	9 (47.4)
Physical Therapy (n = 19)	
Yes	9 (47.4)
No	10 (52.6)
Speech Language Pathology (n = 16)	
Yes	3 (18.8)
No	13 (81.3)
**Functional Impairment (n = 33)**	
Hearing	5 (15.1)
Seeing	11 (33.3)
Learning New Information (*n* = 32)	11 (34.4)
Controlling Behavior	12 (36.4)
Reacting to Sensations	13 (39.4)
Communicating to Others	16 (48.5)
Paying Attention	20 (60.6)
Using Hands to do Activities	20 (60.6)
Remembering information (e.g directions)	21 (63.6)
Managing Emotions (*n* = 32)	21 (68.8)
Moving Around	23 (69.7)
**Health Conditions (n = 33)**	
ASD	1 (3)
Vision	2 (6.1)
Other Impairments (Stroke, Borderline Personality Disorder and Sjogren Autoimmune Disease and Vestibular Deficit)	3 (9)
Developmental Delay	3 (9.1)
Intellectual Delay	3 (9.1)
Hearing	3 (9.1)
Serious Emotional Disturbance	3 (9.1)
ADD	3 (9.1)
Speech or Language	4 (12.1)
TBI	4 (12.1)
Multiple Disabilities	5 (15.2)
Specific Learning Disabilities	6 (18.2)
Health Impairment (e.g. epilepsy/seizures, asthma, cardiac or heart problems, arthritis)	8 (24.2)
Orthopedic/Movement	23 (69.7)

**Figure 1:**
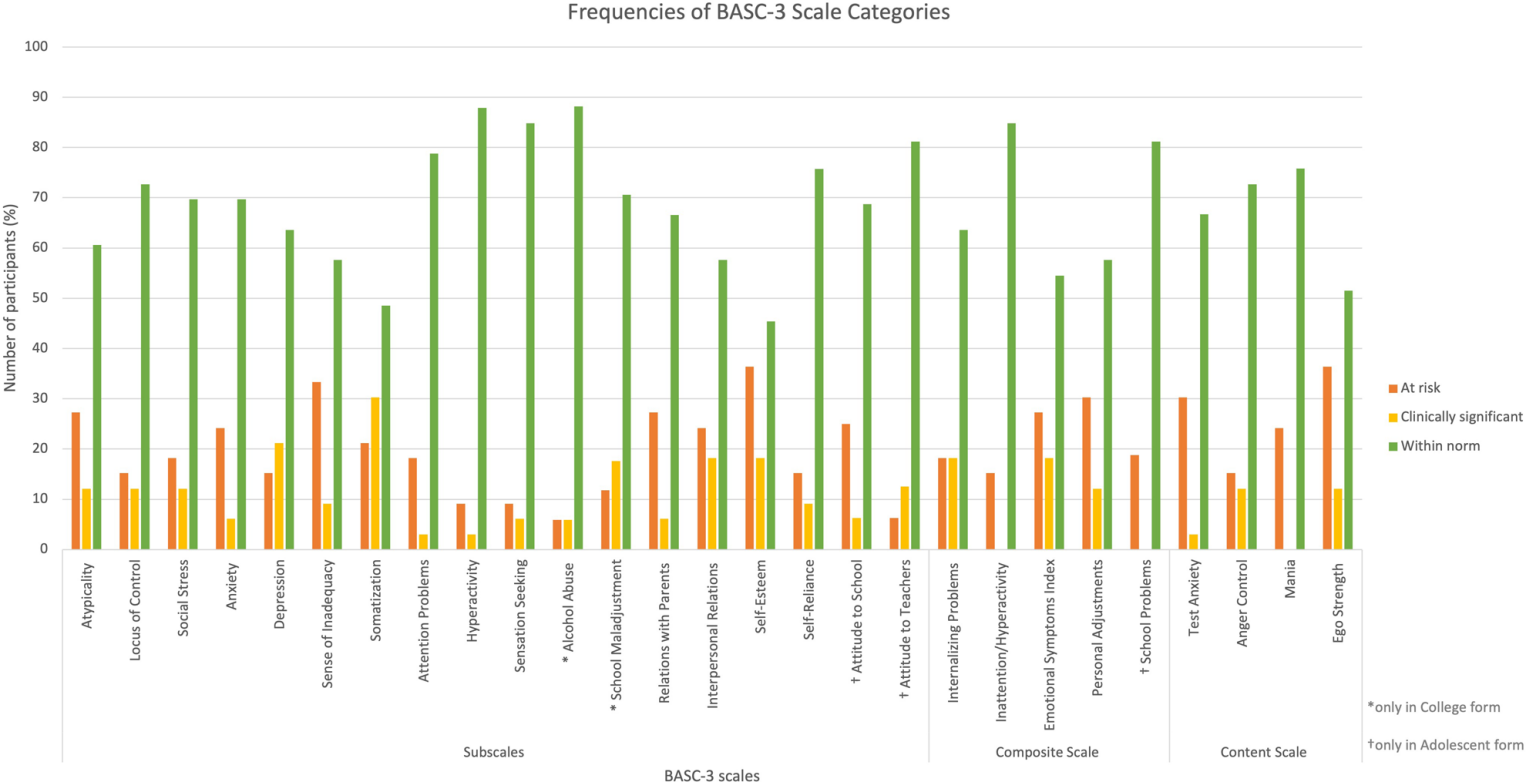
Frequencies of At risk and clinically significant scores for each BASC-3 scale.

Frequency of occurrences of self-reported mental health problems across BASC-3 categories (*n* = 33):

As shown in Figure 1, all BASC-3 scales had at least one participant who fell in the “at risk” category, indicating that all BASC-3 scales representing a range of mental health problems were relevant for youth with disabilities in our studied sample.

### BASC-3 scales falling “within norms” category

For the BASC-3 subscales, the most frequent subscales falling “within norms” were hyperactivity (87.9%) closely followed by sensation seeking (84.8%), while the least common ones were somatization (48.5%) and sense of inadequacy (57.6%). With respect to the composite scales, inattention/hyperactivity (84.8%) followed by internalizing problems (63.6%) ranked most frequently “within norms” while the least common ones were emotional symptoms index (54.5%) and personal adjustments (57.6%). With respect to content scales, mania (75.8%) ranked most frequently “within norms” followed by anger control (72.7%) while ego strength (51.5%) and test anxiety (66.7%) were the least common ones.

### BASC-3 scales falling within “at risk” category

For the BASC-3 subscales, as shown in Figure 1, the most frequent subscales for which participants ranked “at risk” were self-esteem (36.4%) closely followed by sense of inadequacy (33.3%) while the least common ones were hyperactivity (9.1%) and sensation seeking (9.1%). With respect to the composite scales, personal adjustments (30.3%) ranked most frequently “at risk” closely followed by emotional symptoms index (27.3%) while the least common ones were inattention/hyperactivity (15.2%) and internalizing problems (18.2%). With respect to content scales, ego strength (36.4%) ranked most frequently “at risk” followed by test anxiety (30.3%) while anger control (15.2%) and mania (24.2%) were the least common ones.

### BASC-3 scales falling within “clinically significant” category

The most frequent “clinically significant” BASC-3 subscales were somatization (30.3%) followed by depression (21.2%) whereas the least common ones were attention problems (3.0%) and hyperactivity (3.0%). With respect to the composite scales, internalizing problems (18.2%) and emotional symptoms index (18.2%) ranked most frequently “clinically significant” while the least common one was personal adjustments (12.1%). None of the scores pertaining to inattention/hyperactivity fell within the “clinically significant” category. As for the content scales, anger control and ego strength (12.1%) ranked most frequently “clinically significant” while test anxiety (3.0%) was the least common one. None of the scores pertaining to mania fell within the “clinically significant” category.

### BASC-3 scales falling within “at risk and above” category

In total, participants ranked most frequently “at risk and above” for self-esteem (54.5%) closely followed by somatization (51.5%) while least frequently for hyperactivity (12.1%) and sensation seeking (15.2%). With regards to the composite scales, emotional symptoms index (45.5%) closely followed by personal adjustments (42.4%) ranked most frequently “at risk and above” while the least common ones were inattention/hyperactivity (15.2%) and internalizing problems (36.4%). With respect to content scales, ego strength (48.5%) and test anxiety (33.3%) ranked most frequently “at risk and above” while the least common ones were mania (24.2%) and anger control (27.3%).

### Occurrence of mental health (behavioral and emotional) problems across the sexes

Among female participants, atypicality, sense of inadequacy, self-esteem, test anxiety, and ego strength were most frequently ranked “at risk” (45%), while somatization was most frequently ranked “clinically significant” (45%) and “at risk and above” (70%). Within the male participants, emotional symptoms index was the most frequent scale ranked “at risk” (30.8%), while self-esteem was the most frequent subscale ranked “clinically significant” (23.1%) and “at risk and above” (46.2%).

Overall, nine scales indicated a trend with sex, among which three were statistically significant (see Table 3). Female compared to male participants fell in “at risk and above” in eight scales: atypicality, social stress, anxiety, depression, somatization, relationship with parents, internalizing problems, and text anxiety. Among them, a statistically significant trend was observed for three scales including atypicality, somatization and text anxiety. In contrast, males fell in “at risk and above” for sensation seeking scale, yet this association was not statistically significant.

**Table 3:** Associations between BASC-3 scales and sex, number of functional issues and age.

	Sex (Female & Male)		Number of Functional Issues (Higher & Lower)**^c^**		Age (Younger & Older)**^d^**	
	Trend^a^	*P* value**^b^**	Trend	*P* value	Trend	*P* value
**Subscales**						
Atypicality	F > M*	0.003	H > L	0.101		0.71
Locus of Control		0.263	H > L	0.118		0.698
Social Stress	F > M	0.05	H > L	0.057	Y > O	0.257
Anxiety	F > M	0.05	H > L	0.708	Y > O	0.057
Depression	F > M	0.067	H > L*	0.041		0.716
Sense of Inadequacy		0.275	H > L	0.579		0.966
Somatization	F > M*	0.008	H > L*	0.024		0.579
Attention problem		0.202	H > L	0.085		1
Hyperactivity		1		1	Y > O*	0.024
Sensation Seeking	M > F	0.36	L > H*	0.018	Y > O	0.138
Relations with Parents	F > M	0.132	H > L	0.325		0.719
Interpersonal Relations		0.275	H > L*	0.008		0.966
Self-esteem		0.435	H > L	0.611		0.797
Self-reliance		0.431	H > L	0.225		0.416
**Composite Scales**						
Internalizing Problems	F > M	0.067	H > L*	0.041		1
Inattention/Hyperactivity		0.625	H > L*	0.044	Y > O	0.506
Emotional Symptoms Index		0.625	H > L	0.112		0.628
Personal Adjustments		0.515	H > L	0.579		0.653
**Content Scales**						
Test Anxiety	F > M*	0.022	H > L	0.085	Y > O	0.459
Anger control		1	H > L	0.438		0.698
Mania		0.431	H > L	0.688	Y > O*	0.047
Ego Strength		0.353	H > L	0.598		0.881

* Significant for *p* < 0.05.

^a^ Trends indicate those that were “above at risk”. Blank spaces indicate that there is no trend/no association identified.

^b^ Pearson Chi Square test was used when cell count < 5 and Fischer's exact test was used when cell count ≥ 5. .

^c^ Higher (H): ≥ 6 functional issues; Lower (L): < 6 functional issues.

^d^ Younger (Y): < 20 years old; Older (O): ≥ 20 years old.

### Mental health problems across functional issues

Among the sub-group of participants with higher number of functional issues (≥6), atypicality, self-esteem, test anxiety and ego strength were most frequently ranked “at risk” (41.2%), while somatization was most frequently ranked “clinically significant” (47.1%) and “at risk and above” (70.6%). Amongst participants with lower number of functional issues (<6), sense of inadequacy, self-esteem, emotional symptoms index, personal adjustments, and ego strength were most frequently ranked “at risk” (31.3%), while self-esteem was most frequently ranked “clinically significant” (18.8%) and “at risk and above” (50.1%).

In general, the crosstab analysis revealed that descriptively those with greater number of functional issues fell within the “at risk and above” category for most of the BASC-3 scales (20/22). The association between number of functional issues and presence of mental health problems (ranked “at risk and above”) was statistically significant in six scales. Specifically, youth with higher number of functional issues tended to experience depression, somatization, inattention/hyperactivity, problems with interpersonal relations, and to internalize problems, whereas those with lower number of functional issues were significantly more likely to seek sensations.

### Mental health problem across age groups

For younger participants, aged below 20 years, anxiety, personal adjustments, mania, and ego strength were most frequently ranked “at risk” (42.9%), while depression, somatization, self-esteem, and anger control were most frequently ranked “clinically significant” (21.4%), and somatization and self-esteem “at risk and above” (57.1%). Within participants aged 20 and above, self-esteem was most frequently ranked “at risk” (36.8%), while somatization was most frequently ranked “clinically significant” (36.8%) and self-esteem “at risk and above” (52.6%).

Overall, a descriptive trend between the presence of mental health problems and age groups was observed in seven BASC-3 scales, where younger participants (aged below 20 years) were more likely to fall within the “at risk and above” category than the older group (aged 20 years and above). The BASC-3 subscales that showed significant association with age were hyperactivity and mania, revealing that younger participants were significantly more likely to fall within the “at risk and above” category than the older group (see Table 3). A similar trend (yet non-significant) was observed for anxiety, social stress, sensation seeking, inattention/hyperactivity, and test anxiety.

## Discussion

This brief research report provides additional evidence for the occurrences of emerging mental health problems, measured by a comprehensive self-reported assessment (BASC-3), among transition-aged youth with physical disabilities. It also describes these co-occurrences across sex, age, and functional issues. Overall, when looking at the entire sample, “somatization”, “self-esteem”, “depression” and “sense of inadequacy” were the most common areas where youth reported difficulties, falling within the “at risk” or “clinical significant” categories. Similarly, Gorter et al. (22) found that depressive and anxious symptoms were commonly present in adolescents and young adults with cerebral palsy. Another study described a higher risk of depression for adults living with cerebral palsy compared to adults without cerebral palsy (23). Our findings indicated that “somatization”, referring to physical health symptoms related to emotional state, was the most frequent subscale. This is worth reflecting on. It is unclear whether the frequency of somatization was found due to youth's actual emotional state or was observed rather due to the physical discomfort emerging from the complex nature of the youth's physical condition. Thus, further investigation of the origin of somatization is needed. This is of particular importance as previous research indicated that mental health symptoms were associated with fatigue and pain among youth with cerebral palsy (22).

Several associations (or trends) were observed between mental health issues and personal factors with a more pronounced association with complexity of the youth's condition. Specifically, youth with higher number of functional issues were more likely to fall within the “at risk and above” category for most of the BASC-3 scales, except for hyperactivity and sensation seeking. Although these associations were not statistically significant across all scales, it is important to reflect on the identified trends as prior research has shown similar findings. For example, Lindén-Boström & Persson (24) found that youth (13–18 years old) with multiple impairments had a higher probability of developing poorer mental health. This may be explained by the overall lower levels of participation evident in this population (25) as lower rates of participation have been associated with a greater occurrence of mental health problems among youth (26). To decrease the prevalence of mental health issues amongst youth with disabilities, Granlund et al. (27) suggested that interventions should be focused on increasing participation levels, to increase overall well-being and indirectly improve mental health. A study by Anaby et al. (28) demonstrated that after a small group of youth (15–25 years old) participated in a meaningful activity for 8 weeks, at least one component of the affective body function (e.g., anxiety, sense of inadequacy) measured by the BASC-3, showed significant improvement. Thus, to mitigate the occurrence of mental health problems in youth with physical disabilities, further research should conduct larger scale participation-focused interventions for youth with physical disabilities.

Some initial trends were observed across the sexes where nine scales of the BASC-3 showed a trend or an association with sex. Specifically, female adolescents, in comparison to male participants, were significantly more likely to fall with the “at risk and above” category on eight scales including self-esteem, test anxiety, atypicality and somatization. Our findings are similar to what has been establish in literature, showing sex differences for mental health in general (24). Females are more likely to be affected by depression, psychological distress, and anxiety than males depending on the context (29, 30). Furthermore, socio-cultural, psychological, and biological factors can be important but not inclusive factors to explain these disparities (31, 32). Gender-related beliefs and participation in different activities or social roles can also have an incidence on the results (29).

Age also appeared to be an important factor in some of the BASC domains. Results demonstrated that participants in the younger age group ranked more “at risk and above” compared to participants in the older age group across 7 (out of 22) subscales. Similarly, a study by Brossard-Racine et al. (33) found that younger adolescents with cerebral palsy scored higher in most subscales of the Strengths and Difficulties Questionnaire, a screening questionnaire that measures behavioral and emotional problems in children and adolescents, compared to their older peers, which suggests that these problems appeared during earlier childhood (34). These findings, in combination with the findings of the present study, may indicate the value of early intervention in younger adolescents to address existing mental health issues as well as the prevention of the latter to further develop into mental illness. Intervention and prevention of mental health issues can be done through the increase of their participation, as decreased activity participation can lead to higher prevalence of mental health problems in youth with physical disabilities (6, 7, 27). In addition, self-esteem was the most frequent subscale observed to be “at risk” for both the older and younger age groups. This suggests that self-esteem of youth with physical disabilities is affected throughout their adolescence and early adulthood, regardless of the age, which may be due to pre-conceived notions of others towards their disability, as research showed that perceived disability stigma is correlated with self-esteem in adolescents with physical disabilities (35).

Our results, based on the BASC-3 clinical norms, suggest that youth with physical disabilities are at a greater risk of mental health problems, evident in 12% to 54% of our sample (those falling under categories of “at risk” or “clinically significant”) and comparable to emerging findings (15, 17). The findings also shed light on the applicability and uniqueness of the BASC-3 as a comprehensive self-rated measure to assess aspects related to mental health issues in this population. Specifically, it was observed that all scales from the BASC-3 had at least one participant that fell above at risk. This suggests the relevancy of each scale that represents the various mental health problems experienced by our sample group depicted by the BASC-3 and, thus, may be considered for use in future studies.

## Limitations and future directions

Generalizability of the findings towards the larger population of youth with physical disabilities may be impacted by sampling strategy (convenience sampling) as well as inclusion and exclusion criteria, where individuals presenting with an intellectual delay, or a neurodegenerative disorder were excluded. When looking at data analysis, given that sampling distribution has an approximate chi-square distribution, a small sample size can impact accuracy of the approximation (36). Furthermore, 72.7% of the sample data was collected during the COVID-19 pandemic, which may affect findings. Further larger studies considering both adolescents and college-aged young adults are also needed, especially those combining a qualitative element. This may allow for greater insights on co-occurrences between mental health problems and physical disability. Lastly, further studies should include other factors that may explain mental health problems such as participation and social support, factors known to affect youth's well-being (5).

## Data Availability

The datasets presented in this article are not readily available because; The dataset must remain strictly confidential and not be shared in order to respect participants’ preferences. This is particularly important as the dataset includes sensitive information on youth's mental health and involves a relatively small sample size that may be identified. Requests to access the datasets should be directed to dana.anaby@mcgill.ca.
